# Mucosal-associated invariant T cells predict increased acute graft-versus-host-disease incidence in patients receiving allogeneic hematopoietic stem cell transplantation

**DOI:** 10.1186/s12935-022-02703-x

**Published:** 2022-09-30

**Authors:** Zhao Wang, Sudong Zhang, Xiaoyu Zhang, Li Liu, Lukun Zhou, Yuyan Shen, Rongli Zhang, Yi He, Donglin Yang, Erlie Jiang, Xiaoming Feng, Jiaxi Zhou, Tao Cheng, Mingzhe Han, Sizhou Feng

**Affiliations:** 1 State Key Laboratory of Experimental Hematology, National Clinical Research Center for Blood Diseases, Haihe Laboratory of Cell Ecosystem, Institute of Hematology & Blood Diseases Hospital, Chinese Academy of Medical Sciences & Peking Union Medical College, 288 Nanjing Road, Tianjin, 300020 China; 2 Department of Hematology, Tianjin First Central Hospital, School of Medicine, Nankai University, Tianjin, 300192 China

**Keywords:** MAIT cell, Hematopoietic cell transplantation, Graft versus host disease, Cytokines

## Abstract

**Background:**

Mucosal-associated invariant T (MAIT) cells are innate-like T cells, some studies have reported that the number of circulating MAIT cells reduced in patients with acute graft-versus-host-disease (aGVHD) development. However, the role of donor MAIT cells on aGVHD development and subsequent functional change still remain unclear.

**Methods:**

The study recruited 86 patients with hematological malignancies who underwent allogeneic hematopoietic cell transplantation (HCT) from May 1, 2018 to June 30, 2019. MAIT cells, their subset, and cytokine levels were measured by flow cytometry. Gray’s test was used to assess the impact of graft MAIT cell proportion and number on aGVHD incidence. The Cox proportional hazard model was used in the multivariate analysis. The comparison for continuous variables was assessed using Mann–Whitney analysis. RNA-sequencing was performed to investigate the possible molecular pathway changes.

**Results:**

Our study showed that the proportion of MAIT cells in grafts was not different from normal controls, but the CD4/8 subsets were altered. Taking the median of the proportion and number of MAIT cells in the graft as the threshold, the results showed that the incidence of grade B-D aGVHD in patients with MAIT cell proportion ≥ 3.03% was significantly higher than that in patients with MAIT cell proportion < 3.03% (56.3%, 95% CI 37.1–71.2 versus 23.1%, 95% CI 13.8–46.2; *P* = 0.038).The number of MAIT cells in the graft was not associated with aGVHD development (*P* = 0.173), however, when the graft contained more CD4 positive, CD8 positive, and CD4/CD8 double-positive MAIT cells, the incidence of aGVHD was significantly increased (*P* = 0.019, *P* = 0.035 and *P* = 0.027, respectively). Besides, reduced frequencies and counts of circulating MAIT cells were observed in patients with aGVHD when compared to patients without aGVHD, accompanied by enhanced production of Tumor necrosis factor-α, Interferon-γ and upregulated programmed death-1, CXC Chemokine Receptor-6 (CXCR6) and CD38 expression. Gene set enrichment analysis of MAIT cell RNA-seq data showed interferon-α response pathway upregulated in aGVHD patients when compared with patients without aGVHD and healthy controls.

**Conclusions:**

Our study shows that MAIT cells in grafts and peripheral blood are both closely related to the aGVHD development post allogeneic HCT. Interferon-α response pathway perhaps is a critical regulation mechanism for the MAIT cell involvement in aGVHD development.

**Supplementary Information:**

The online version contains supplementary material available at 10.1186/s12935-022-02703-x.

## Background

Acute graft-versus-host disease (aGVHD) is a significant cause of morbidity and transplantation-related mortality in patients receiving allogeneic hematopoietic cell transplantation (HCT). It has been established that antigen-presenting cells, conventional T cells, and some other immune effector cells participate in this complex interaction between innate and adaptive immune systems [1]. Pro-inflammatory cytokines released by T cells could amplify this immune response and result in tissue damage [2].

Mucosal-associated-invariant T (MAIT) cells are a group of highly evolutionarily conserved innate-like T cells with an invariant T cell receptor Va7.2–Ja33 chain in humans. After birth, MAIT cells migrate to peripheral tissues including peripheral blood, intestine, lymph nodes, and liver [3]. These non-conventional T cells only recognize vitamin B2- and B9-derived ligands, which are produced by a range of bacteria or yeasts [4] and are presented by major histocompatibility class I related molecules [5, 6]. The MAIT cells could also respond to cytokine signals in a T cell receptor (TCR) independent manner [7]. Upon activation, MAIT cells could secrete mass pro-inflammatory cytokines including tumor-necrosis-factor-alpha (TNF-α), interferon-gamma (IFN-γ), interleukin 17 (IL-17), and cell lysis mediators like granzyme-B and perforin [8]. Thus far, it is well acknowledged that MAIT cells are involved in several infections such as tuberculosis, cholera [9] and fungal infections [10–12] and autoimmune diseases such as systemic lupus erythematosus, multiple sclerosis, and inflammatory bowel diseases [13–15].

In patients receiving allogeneic HCT, early reconstitution of MAIT cells can be observed [16]. Recently, some studies have reported the number of MAIT cells in peripheral blood significantly reduced in GVHD patients [17, 18]. However, the impact of MAIT cells in grafts on aGVHD development and subsequent functional changes in aGVHD patients are still largely unknown. Here, we longitudinally analyzed the frequency and function status of MAIT cells in grafts and peripheral blood samples from recipients receiving allogeneic HCT. The investigation showed a high frequency of MAIT cells in grafts correlated to increased incidence of aGVHD post allogeneic HCT, accompanied with a significant numerical reduction and functional activation of MAIT cells in peripheral blood from recipients with aGVHD development. Gene set enrichment analysis (GSEA) of RNA-sequencing data suggested a critical role of the interferon-alpha response of MAIT cells in aGVHD development.

## Methods

### Patients and controls

The study prospectively recruited 86 patients who underwent allogeneic HCT in the Blood Diseases Hospital, Chinese Academy of Medical Sciences from May 1, 2018 to June 30, 2019. Patients eligible for this analysis included all consecutive adult and pediatric recipients who achieved donor-derived neutrophil engraftment after allogeneic HCT. One patient who experienced engraftment failure was excluded. Peripheral blood samples were collected from recipients at day 15, 30 (± 5), 60 (± 5), and 90 (± 5) post allogeneic HCT and peripheral blood samples from healthy subjects as the normal control. For cytokine analysis and RNA-sequencing (RNA-seq), peripheral blood samples were collected when patients were diagnosed with aGVHD, and samples were also collected from patients who did not develop aGVHD at the same time point and used as controls. Graft samples transfused into these recipients were collected from the abandoned testing samples. All blood samples were then examined using flow cytometry and RNA-seq using standard protocols. This study was approved by the Ethics Committee of Blood Disease Hospital, Chinese Academy of Medical Sciences (KT2018104-EC-2). Written informed consent was acquired from all patients and healthy subjects.

### Flow cytometry analysis and MAIT cell sorting

All the peripheral blood and graft samples were purified by density gradient centrifuge using Ficoll–Paque to achieve mononuclear cells. The MAIT cells and subgroups were examined using the following antibodies: anti-CD3-APC-Cy7, anti-TCRγδ-FITC, anti-CD161-Percp-Cy5.5, anti-TCRVα7.2-APC, anti-CD4-PE, anti-CD8-PE-Cy7, anti-CXCR6-PE, anti-PD1-PE, and anti-CD38-PE-Cy7. Mononuclear cells (1 × 10^6^) were incubated in complete media for intracellular cytokine staining. All samples were acquired on an LSRFortessa flow cytometer. Data analysis was performed using the FlowJo Version 7.6 software.

For peripheral blood MAIT cell sorting, samples obtained from patients and healthy controls were purified by density gradient centrifuge. Mononuclear cells were stained with surface antibodies (anti-CD3-APC-Cy7, anti-TCRγδ-FITC, anti-CD161-Percp-Cy5.5, anti-TCRVα7.2-APC). Then, the cells were sorted immediately using an FACSAria III cell sorter (BD Bioscience, Franklin Lake, NJ, USA), selecting CD3^+^ TCRγδCD161^+^TCR-Va7.2^+^ cells.

### In vitro functional analysis

Phorbol myristate acetate (PMA), ionomycin (IM) and 1 μL monensin were added to the media at the beginning of incubation. After incubation for 4 h, cells were aspirated and stained with the surface markers, followed by fixation in Cytofix/Cytoperm and permeabilized with Perm/Wash solution according to the manufacturer’s introductions. The cells were then stained with cytokine antibodies.

### RNA-seq

RNA-seq was performed on MAIT cells from peripheral blood samples as previously described [19]. Each patient collected 1 × 10^4^ MAIT cells for sequencing, and totally, ten samples were sequenced, including four samples from patients with aGVHD, three from patients without aGVHD, and three from healthy controls. RNA concentration was measured using Qubit RNA Assay Kit (Life Technologies, CA, USA), and RNA integrity was assessed using Bioanalyzer 2100 system (Agilent Technologies, CA, USA) with RIN > 6.5.The libraries were generated using the NEBNext UltraTM RNA Library Prep Kit for Illumina (New England Biolabs, Ipswich, MA, USA) following the manufacturer’s recommendations. The clustering of the samples was performed on a cBot Cluster Generation System using TruSeq PE Cluster Kit v3-cBot-HS (Illumina), then the library preparations were sequenced on an Illumina Hiseq platform with a target depth of 3 million reads. The data are available at Gene Expression Omnibus (GEO) with the accession number of GSE157959.

### Study definition and statistical analysis

aGVHD was diagnosed clinically according to the International Bone Marrow Transplant Registry [20]. Bacterial infections were defined as: positive clinical symptoms of bacterial infection and positive culture results, including blood, sputum, sterile tissue, bronchoalveolar lavage fluid and urinary fluid. Fungal infections were defined as: diagnosis of proven and probable fungal infection following guidelines of Infectious Diseases Society of America [21, 22]. Viral infections were defined as: cytomegalovirus or Epstein–Barr virus > 400 copies/mL in plasma using quantitative polymerase chain reaction. The cumulative incidence of aGVHD was estimated and compared using Gray’s test. The multivariate Cox regression model was used to investigate the association between MAIT cells and the hazard of aGVHD. All factors found to influence aGVHD in univariate analysis with *P* < 0.20 were included in the Cox proportional hazard model. Correlations were analyzed using the Spearman’s rank correlation test. A significant difference for continuous variables was assessed using Mann–Whitney analysis. *P* ≤ 0.05 was considered significant. All Statistical analysis was performed using SPSS 12, except that for Gray’s test, which was performed using R 3.6.1.

## Results

### Patient and transplantation characteristics

The characteristics of all 86 patients in the study are shown in Table 1. The median age of recipients at the time of allogeneic HCT was 37 (range 7–63) years. 41 patients (47.6%) received allogeneic HCT for acute myeloid leukemia, 19 (22.1%) for acute lymphoid leukemia, 25 (29.1%) for myelodysplastic syndrome, and one (1.2%) for primary myelofibrosis. All patients received myeloablative conditioning, either a regimen based on busulfan and cyclophosphamide (n = 69) or a regimen based on total body irradiation and cyclophosphamide (n = 17). 45 patients received HLA-matched sibling donor grafts, 37 received haploidentical donor grafts, and only four received HLA-matched unrelated donor grafts. The GVHD prophylaxis regimens included cyclosporine A/methotrexate (n = 26), cyclosporine A/methotrexate/mycophenolate mofetil (n = 33), and tacrolimus/mycophenolate mofetil (n = 27). Anti-thymocyte globulin (ATG) was used as an alternative approach to the post-transplant cyclophosphamide (PT-Cy) for in vivo T cell depletion in haploidentical donor transplantation, HLA matched unrelated donor transplantation and sibling donor transplantation with the recipients’ age older than 45 years. Totally, 64 patients of the enrolled patients used ATG. Of all the donors, 35 cases were female donor, including 21 cases of female to male and 14 cases of female to female. CMV-seropositive donor to CMV-seropositive recipients were the most common donor–recipient CMV serostatus (n = 81). The median number of infused total mononuclear cells and CD34^+^ cells were 9.7 × 10^8^/kg (range 5.2–18.5) and 3.1 × 10^6^/kg (range 1.6–6.7), respectively. None of the patients underwent ex vivo T cell depletion. In our study, 44 cases developed aGVHD in 100 days with a cumulative incidence of 50.6% (95% confidence interval [CI] 39.9–61.2%), including 15 cases of grade A aGVHD, 17 cases of grade B aGVHD, six cases each of grade C aGVHD and grade D aGVHD; Of these patients who developed aGVHD, 21 cases had skin involvement, 14 had liver involvement, and 17 had intestinal involvement; 13 patients had ≥ 2 organ involvement. The median onset time of aGVHD was 31 (range 11–58) days post graft transfusion.

**Table 1:** Patient characteristics enrolled in this study

Characteristic	Value
Median age (range), y	37 (7–63)
Male, N (%)	47 (54.6)
Diagnosis, N (%)	
AML	41 (47.6)
ALL	19 (22.1)
MDS	25 (29.1)
PMF	1 (1.2)
Conditioning regimens, N (%)	
Bu + Cy + Flu + Ara-C	69 (80.2)
TBI + Cy + Flu + Ara-C	17 (19.8)
Donor type, N (%)	
HLA-matched sibling	45 (52.3)
HLA-matched unrelated	4 (4.7)
HLA-haploidentical	37 (43.0)
GVHD prophylaxis	
CSA + MTX	26 (30.2)
CSA + MMF + MTX	33 (38.3)
Tacrolimus + MTX	27 (31.3)
ATG, N (%)	
With ATG	64 (74.4)
Without ATG	22 (25.6)
Donor/recipient gender	
Female to male	21 (24.4)
Female to female	14 (16.3)
Male to female	25 (29.1)
Male to male	26 (30.2)
CMV serostatus	
D/R	0
D/R +	3 (3.5)
D + /R +	81 (94.2)
D + /R	2 (2.3)
Median infused MNC dose (range)	9.7 × 10^8^/kg (5.2–18.5)
Median infused CD34^+^ dose (range)	3.1 × 10^6^/kg (1.6–6.7)

D− /R− donor CMV seronegative/recipient CMV seronegative, D− /R+ donor CMV seronegative/recipient CMV seropositive, D+ /R+ donor CMV seropositive/recipient CMV seropositive, D+ /R−  = donor CMV seropositive/recipient CMV seronegative

AML acute myeloid leukemia, ALL acute lymphoid leukemia, MDS myelodysplastic syndrome, AA aplastic anemia, PMF primary myelofibrosis, Bu busulfan, Cy cyclophosphamide, TBI total body irradiation, Flu fludarabine, Ara-C cytarabine, CSA cyclosporine A, MTX methotrexate, MMF mycophenolate mofetil, ATG anti-thymocyte globulin, MNC mononuclear cells, CMV Cytomegalovirus

### Mobilization characteristics of MAIT cells in grafts

**Fig. 1:**
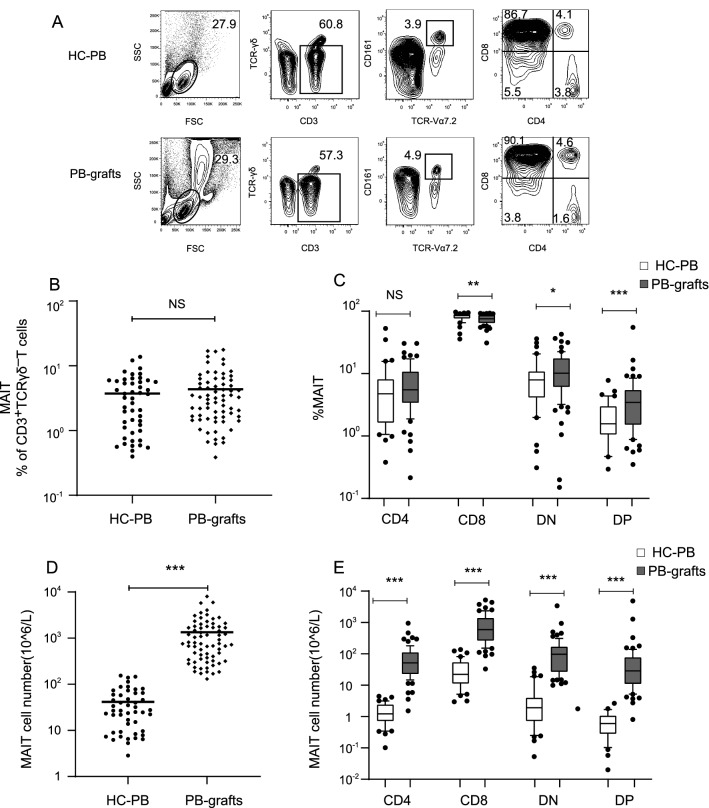
Mucosal-associated-invariant T (MAIT) cells mobilization characteristics in PB-grafts. **A** Gating strategy and a representative example of flow cytometric analysis of one peripheral blood graft (PB-graft) sample and one healthy subject peripheral blood (HC-PB) sample. **B** The frequency of MAIT cells in PB-grafts (n = 69) and HC-PB (n = 48). **C** Proportion of CD4, CD8, DN, DP subsets in PB-grafts (n = 66) and healthy controls (n = 40). **D** The number of MAIT cells in PB-grafts (n = 69) and HC-PB (n = 48). **C** number of CD4, CD8, DN, DP subsets in PB-grafts (n = 66) and healthy controls (n = 40). *P-value < 0.05; **P-value < 0.01; ***P-value < 0.001; NS: no significance

Among the enrolled 86 patients, 69 peripheral blood stem cell graft (PB-graft) samples mobilized by granulocyte-colony-stimulating factors were collected. MAIT cells were identified using flow cytometry and defined as CD3^+^TCRγδVα7.2^+^CD161^+^ cells as previously described [23] (Fig. 1A). According to our result, no significant differences had been found in MAIT cell proportions between PB-grafts (n = 69) and blood samples from healthy donors (n = 48) (*P* = 0.87, Fig. 1B). According to the expression levels of CD4/8 on the surface of MAIT cells, we then subdivided MAIT cells into four subsets: CD4 positive, CD8 positive, CD4/CD8 double negative (DN), and CD4/CD8 double positive (DP) subsets. Results showed that the percentage of CD8 positive MAIT cells in PB-grafts (n = 66, median percentage: 75.1%) was significantly lower than that in healthy donors (n = 40, median percentage: 81.1%, *P* = 0.006). Otherwise, the frequency of DN-MAIT cells (median percentage: 12.4%) and DP-MAIT cells (median percentage: 4.7%) from PB-grafts were both significantly higher than those from healthy controls (DN: median percentage 9.6%, *P* = 0.046; DP: median percentage 2.1%, *P* = 0.001; Fig. 1C), which indicated that the transfused MAIT cell subpopulations in grafts had transformed after mobilization. In addition, the number of MAIT cells in the graft was significantly higher than that in the healthy control (134.8 × 10^7^/L vs. 4.1 × 10^7^/L, *P* < 0.001, Fig. 1D); the number of each CD4/8 subset was also significantly higher than that in the control group (CD4 + : 91.3 × 10^6^/L vs. 1.6 × 10^6^/L, *P* < 0.001; CD8 + : 963.2 × 10^6^/L vs. 36.5 × 10^6^/L, *P* < 0.001; DN: 178.0 × 10^6^/L vs. 4.8 × 10^6^/L, *P* < 0.001; DP: 46.1 × 10^6^/L vs. 0.73 × 10^6^/L, *P* < 0.001; Fig. 1E).

### Factors affecting the reconstitution of MAIT cells post allogeneic HCT

**Fig. 2:**
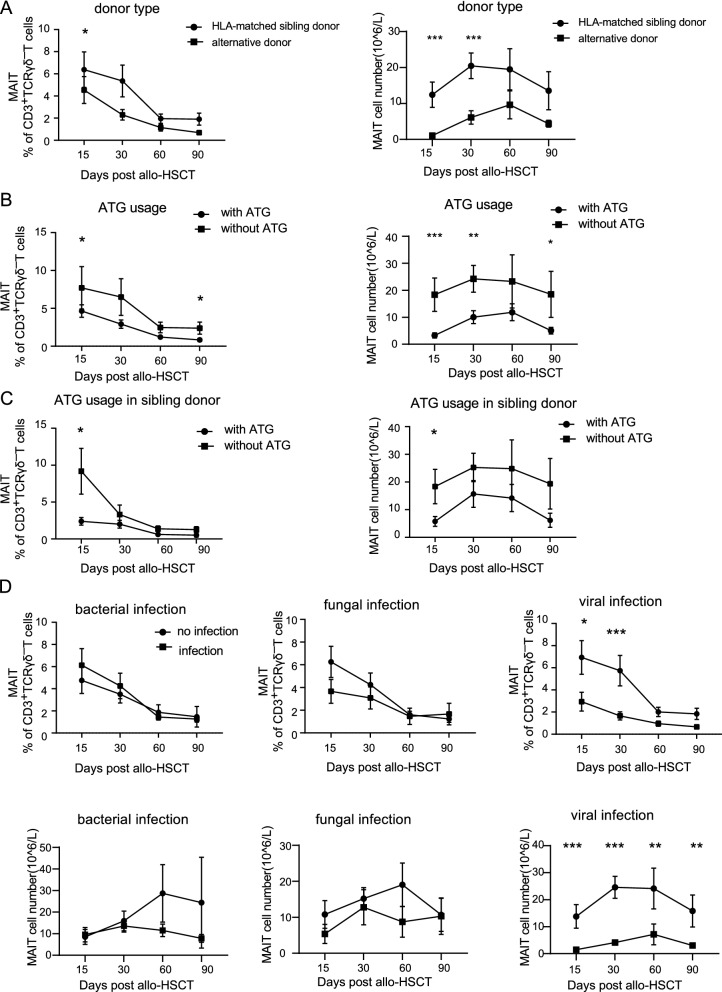
Factors that affect the reconstitution of MAIT cells post allogeneic HCT. **A** Mucosal-associated-invariant T cell frequencies and counts in peripheral blood from recipients receiving HLA-matched sibling donor transplantation (n = 45) and alternative donor transplantation (n = 41). **B** MAIT cell frequencies and counts from recipients receiving transplantation with ATG (n = 64) and without ATG (n = 22) in the conditioning regimes. **C** MAIT cell frequencies and counts from recipients receiving HLA-matched sibling donor transplantation with ATG (n = 23) and without ATG (n = 22) in the conditioning regimes. **D** MAIT cell frequencies and counts from recipients existing with or without bacterial infection (n = 63 and 23, respectively); fungal infection (n = 17 and 69, respectively) and viral infection (n = 34 and 52, respectively). Mann–Whitney test was used to assess significant differences between groups. Mean ± standard error is shown. *P-value < 0.05; **P-value < 0.01; ***P-value < 0.001

We also analyzed the clinical factors that may affect the reconstitution of MAIT cells post allogeneic HCT, and the results showed that the application of ATG, type of donor, and viral infection (Cytomegalovirus and Epstein–Barr virus) significantly affected the early reconstitution of MAIT cells. Patients receiving matched-sibling donor grafts had a better MAIT reconstitution compared with those receiving alternative donor grafts (Fig. 2A). In addition, application of ATG in conditioning regimens could significantly reduce the proportion and number of MAIT cells in the recipients’ peripheral blood, and this effect could still be observed at day 90, post graft transfusion (Fig. 2B). In order to avoid the effect of donor type on MAIT cell reconstitution, we further investigated the ATG usage on the proportion and number of MAIT cells in recipients receiving HLA-matched sibling donor transplantation, and the effect of ATG on MAIT cells can still be observed (Fig. 2C). For the factor of infection, we found that viral infections were significantly related to the decrease in the proportion and number of MAIT cells post allogeneic HCT, while bacterial infections and fungal infections had no obvious correlation with MAIT cell reconstitution (Fig. 2D).

### Higher MAIT cells proportion in grafts predicted higher risk of aGVHD

As it has been reported that MAIT cells in recipients are partly derived from the transfer of MAIT cells in the grafts and are related to the GVHD occurrence post allogeneic HCT [17], we further investigated whether MAIT cell frequency and the absolute number in the grafts could predict the incidence of aGVHD post allogeneic HCT.

**Fig. 3:**
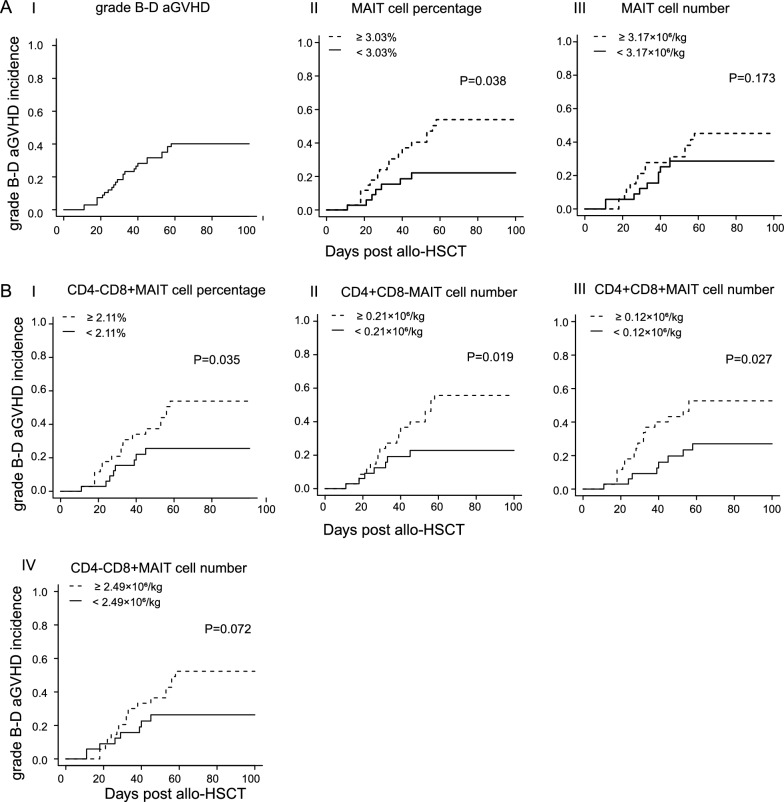
MAIT cell proportion and number in grafts predicted grade (**B**–**D**) aGVHD development. (AI) Cumulative incidence of grades **B**‒**D** aGVHD in the entire cohort (n = 69); grades **B**‒**D** aGVHD incidence according to (AII) MAIT cell proportion; (AIII) MAIT cell number; (BI) CD4-CD8 + MAIT cell proportion; (BII) CD4 + CD8-MAIT cell number; (BIII) CD4 + CD8 + MAIT cell number and (BIV) CD4-CD8 + MAIT cell number in PB-grafts. Gray’s test was used to compare the aGVHD cumulative incidence between groups

The cumulative incidence of grade B-D aGVHD for the entire cohort was 40.8% (95% CI 29.5–53.9) at day 100 post allogeneic HCT (Fig. 3A). The median MAIT cell percentage in PB-grafts was 3.03% (interquartile range 1.63–6.18%). We used this value as a threshold to divide patients into two subgroups, MAIT cell percentage ≥ 3.03% group (interquartile range 3.49–12.6%) and MAIT cell percentage < 3.03% group (interquartile range 0.72–2.23%), to evaluate the impact of MAIT cell frequency on the incidence of grades B‒D aGVHD. Results showed that the cumulative incidence of B‒D aGVHD at day 100 significantly increased in patients receiving grafts with MAIT cell percentage ≥ 3.03% than that with percentage < 3.03% (56.3%, 95% CI 37.1–71.2 versus 23.1%, 95% CI 13.8–46.2; *P* = 0.038; Fig. 3A). We additionally found that a higher proportion of CD4-CD8 + MAIT cells in the grafts were significantly associated with an increased incidence of B‒D aGVHD post allogeneic HCT (*P* = 0.035, Fig. 3B, Table 2). Besides, we further used the median absolute number of MAIT cells (3.17 × 10^6^/kg, interquartile range 1.56–7.48 × 10^6^/kg) as a threshold to evaluate the relationship between MAIT cell number in grafts and incidence of B‒D aGVHD. The cumulative incidence aGVHD in patients with MAIT cell number ≥ 3.17 × 10^6^/kg (interquartile range 4.36–14.83 × 10^6^/kg) was 51.1% (95% CI 35.8–68.9) versus 26.6% (95% CI 14.6–47.2) in patients with MAIT cell number < 3.17 × 10^6^/kg (interquartile range: 0.77–2.80 × 10^6^/kg); but no statistical difference was drawn (Fig. 3A, Table 2; *P* = 0.173), According to the analysis of CD4/8 MAIT cell subsets, it showed that more CD4CD8^+^, CD4^+^CD8, CD4^+^CD8^+^MAIT cells in the grafts indicated an increased incidence of B‒D aGVHD, yet, the CD4CD8MAIT cell number had no impact on aGVHD development (Fig. 3BII–IV, Table 2). We also tried several other cell subsets in PB-grafts, including CD4^+^ T cells, CD8^+^ T cells, CD4^+^CD25^+^T cells, Natural killer cells, and B cells. Using a strategy similar to MAIT cells, the median proportion of each cell population was used as a threshold to divide them into groups with higher proportions and those with lower proportions. No significant impacts were found in these cell subsets on B‒D aGVHD incidence (data are shown in Additional file 1).

**Table 2:** Univariate analysis of factors associated with aGVHD development in 69 patients

Variable	N	aGVHD incidence % (95% CI)	P-value
Patient age, y			
≥ 50	12	46.7 (15.3–70.3)	0.652
< 50	57	20.2 (11.3–34.7)	
Donor			
HLA-matched sibling	36	28.6 (10.9–39.8)	0.031
Alternative donors	33	53.3 (31.5–73.2)	
Conditioning regimens			
Bu + Cy + Flu + Ara-C	58	35.9(21.2–47.7)	0.761
TBI + Cy + Flu + Ara-C	11	45.5 (13.7–68.7)	
ATG			
Yes	50	38.6 (22.1–51.8)	0.805
No	19	37.2 (12.0–54.8)	
GVHD prophylaxis			
CSA + MTX(± MMF)	49	43.4 (30.1–59.2)	0.156
Tacrolimus + MTX	20	23.7 (10.4–51.3)	
Infused MNC dose			
≥ 10 × 10^8^/kg	35	36.6 (17.0–51.0)	0.797
< 10 × 10^8^/kg	34	38.9 (19.4–53.5)	
CD4 + T cell percentage, %			
≥ 36.3	35	35.8 (22.3–54.9)	0.458
< 36.3	34	45.6 (29.2–65.8)	
CD8 + T cell percentage, %			
≥ 28.6	35	39.4 (24.3–58.7)	0.758
< 28.6	34	41.6 (26.7–61.1)	
CD19 + B cell percentage, %			
≥ 16.2	35	37.5 (22.4–57.2)	0.479
< 16.2	34	43.6 (28.4–61.9)	
NK cell percentage, %			
≥ 10.3	35	46.4 (27.9–61.9)	0.596
< 10.3	34	33.5 (19.4–53.6)	
CD4 + CD25 + T cell percentage, %			
≥ 0.24	35	47.2 (36.6–66.0)	0.809
< 0.24	34	33.7 (19.9–53.5)	
MAIT cell percentage, %			
≥ 3.03	35	56.3 (37.1–71.2)	0.038
< 3.03	34	23.1 (13.8–46.2)	
CD4-CD8 + MAIT cell percentage, %			
≥ 2.11	35	54.1 (37.9–72.3)	0.035
< 2.11	34	25.9 (13.8–45.3)	
CD4 + CD8-MAIT cell percentage, %			
≥ 0.16	35	47.9 (28.4–66.3)	0.302
< 0.16	34	32.6 (18.8–51.8)	
CD4 + CD8 + MAIT cell percentage, %			
≥ 0.09	35	43.8 (28.7–62.3)	0.502
< 0.09	34	37.7 (22.8–57.6)	
CD4-CD8-MAIT cell percentage, %			
≥ 0.31	35	44.1 (29.2–62.9)	0.548
< 0.31	34	36.2 (22.0–55.9)	
CD3 + T cell dose			
≥ 1.31 × 10^8^/kg	35	43.7 (28.9–63.6)	0.679
< 1.31 × 10^8^/kg	34	37.2 (22.9–56.2)	
CD4 + T cell dose			
≥ 7.32 × 10^7^/kg	35	35.3 (21.6–54.2)	0.408
< 7.32 × 10^7^/kg	34	45.4 (26.4–60.9)	
CD8 + T cell dose			
≥ 5.73 × 10^7^/kg	35	41.6 (25.5–58.7)	0.967
< 5.73 × 10^7^/kg	34	41.1 (21.8–56.5)	
NK cell dose			
≥ 2.01 × 10^7^/kg	35	47.1 (36.8–66.5)	0.354
< 2.01 × 10^7^/kg	34	34.2 (20.5–53.7)	
B cell dose			
≥ 3.11 × 10^7^/kg	35	34.8 (20.9–66.5)	0.287
< 3.11 × 10^7^/kg	34	46.3 (26.8–65.8)	
CD4 + CD25 + cell dose			
≥ 6.45 × 10^5^/kg	35	31.7 (18.8–50.9)	0.115
< 6.45 × 10^5^/kg	34	49.8 (33.3–68.8)	
MAIT cell dose			
≥ 3.17 × 10^6^/kg	35	51.1 (35.8–68.9)	0.173
< 3.17 × 10^6^/kg	34	26.6 (14.6–47.2)	
CD4-CD8 + MAIT cell dose			
≥ 2.49 × 10^6^/kg	35	52.7 (36.6–65.8)	0.072
< 2.49 × 10^6^/kg	34	26.5 (14.2–46.3)	
CD4 + CD8-MAIT cell dose			
≥ 0.21 × 10^6^/kg	35	56.2 (40.0–73.8)	0.019
< 0.21 × 10^6^/kg	34	22.9 (11.8–42.4)	
CD4 + CD8 + MAIT cell dose			
≥ 0.12 × 10^6^/kg	35	53.1 (37.0–71.1)	0.027
< 0.12 × 10^6^/kg	34	27.3 (14.6–47.2)	
CD4-CD8-MAIT cell dose			
≥ 0.41 × 10^6^/kg	35	41.4 (26.3–60.3)	0.756
< 0.41 × 10^6^/kg	34	39.1 (24.4–58.5)	

*Bu* Busulfan, *Cy* Cyclophosphamide, *TBI* Total body irradiation, *Flu* Fludarabine, *Ara-C* Cytarabine, *ATG* Anti-thymocyte globulin, *CSA* Cyclosporine A, *MTX* Methotrexate, *MMF* Mycophenolate mofetil, *MNC* Mononuclear cells

Alternative donors: including 30 haploidentical donors and 3 HLA-matched unrelated donors

We further performed univariate and multivariate analyses to better explore the risk factors affecting the occurrence of GVHD post allogeneic HCT. Therefore, 69 patients, who obtained the MAIT cell frequencies both in the transfused graft samples and PB samples post allogeneic HCT were enrolled. In addition to the above-mentioned MAIT cell proportion and number in grafts that had a significant impact on the incidence of B‒D aGVHD (Fig. 3), univariate analysis also identified that donor type significantly associated with aGVHD development. The incidence of grade B-D aGVHD in patients receiving HLA-matched sibling donor grafts (28.6%, 95% CI 10.9–39.8) was significantly lower than that in patients receiving alternative donor grafts (53.3%, 95% CI 31.5–73.2; *P* = 0.031, Table 2). Factors influencing aGVHD incidence with *P* < 0.20 in the univariate analysis were included in a multivariate analysis. Since ATG has clearly been reported to have an impact on the occurrence of GVHD, this study also included ATG usage in the multivariate analysis; however, the *P* value of the ATG usage was above 0.2. In total, the following factors were included in the multivariate analysis: donor type, MAIT cell percentage, MAIT cell number, GVHD prophylaxis, and ATG usage. We found that MAIT cell frequency in PB-grafts was still an independent factor for aGVHD occurrence. The aGVHD incidence significantly decreased in patients receiving grafts with MAIT cell percentage < 3.03% when compared with MAIT cell percentage ≥ 3.03% (hazard ratio [HR] = 0.36; 95% CI 0.15–0.88; *P* = 0.025; Table 3). Alternative donor graft was another independent factor associated with increased aGVHD incidence (hazard ratio [HR] = 2.25; 95% CI 1.01–5.05; *P* = 0.048; Table 3). No impact was observed for ATG usage, MAIT cell number, or GVHD prophylaxis on the occurrence of aGVHD (ATG usage: *P* = 0.367; MAIT cell number: *P* = 0.604; GVHD prophylaxis: *P* = 0.839).

**Table 3:** Multivariate analysis of factors on aGVHD incidence

Covariate	Hazard ratio (95% CI)	P-value
Donor type		
HLA-matched sibling	Reference	0.048
Alternative donors	2.25 (1.01–5.05)	
MAIT cell percentage, %		
≥ 3.03	Reference	0.025
< 3.03	0.36 (0.15–0.88)	

### Impaired circulating MAIT cell reconstitution was associated with increased risk of aGVHD

**Fig. 4:**
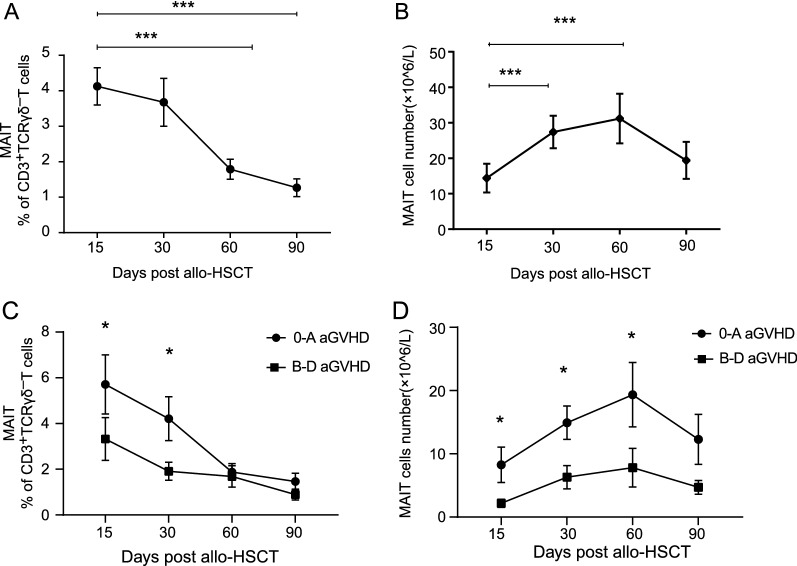
Reduced circulating MAIT cells proportion and number in aGVHD recipients. **A**, **B** MAIT cell early reconstitution in the entire cohort (n = 86). **C**, **D** circulating MAIT cell proportion and number in recipients with grades **B**‒**D** aGVHD in comparison with grades 0‒A aGVHD post allogeneic HCT. Mean ± standard error is shown. Mann–Whitney test was used to assess significant differences between groups. *P-value < 0.05; **P-value < 0.01; ***P-value < 0.001

We further investigated the reconstitution of MAIT cells after allogeneic HCT. The frequencies of circulating MAIT cells at days 15 and 30 post allogeneic HCT were similar, whereas it significantly decreased at day 60 compared to that in patients at day 15, without prominent recovery till day 90 (day 60: 1.78% vs. 3.81%, *P* < 0.0001; day 90: 1.26% vs. 3.81%; *P* < 0.0001, Fig. 4A). However, the absolute number of MAIT cells showed different trend lines. The absolute number of circulating MAIT cells were at the lowest point at day 15 post allogeneic HCT, followed with a significant recovery at day 30 (7.23 × 10^6^/L vs. 12.98 × 10^6^/L, *P* < 0.0001; Fig. 4B).

Our results further identified that both frequency and absolute number of MAIT cells at day 15 in patients with B‒D aGVHD were significantly lower than that in patients without aGVHD (3.32% vs. 5.71%, *P* = 0.327, and 2.18 × 10^6^/L vs. 8.27 × 10^6^/L, *P* = 0.048; Fig. 4C, D). Both frequency and number of MAIT cells at day 30 in patients with B‒D aGVHD (average percentage: 1.91%, average cell number: 6.31 × 10^6^/L) were still lower than those in patients without aGVHD (average percentage: 4.21%, *P* = 0.046; average cell number: 14.92 × 10^6^/L, *P* = 0.024, Fig. 4C, D).

### Function evaluation of MAIT cells in aGVHD recipients

**Fig. 5:**
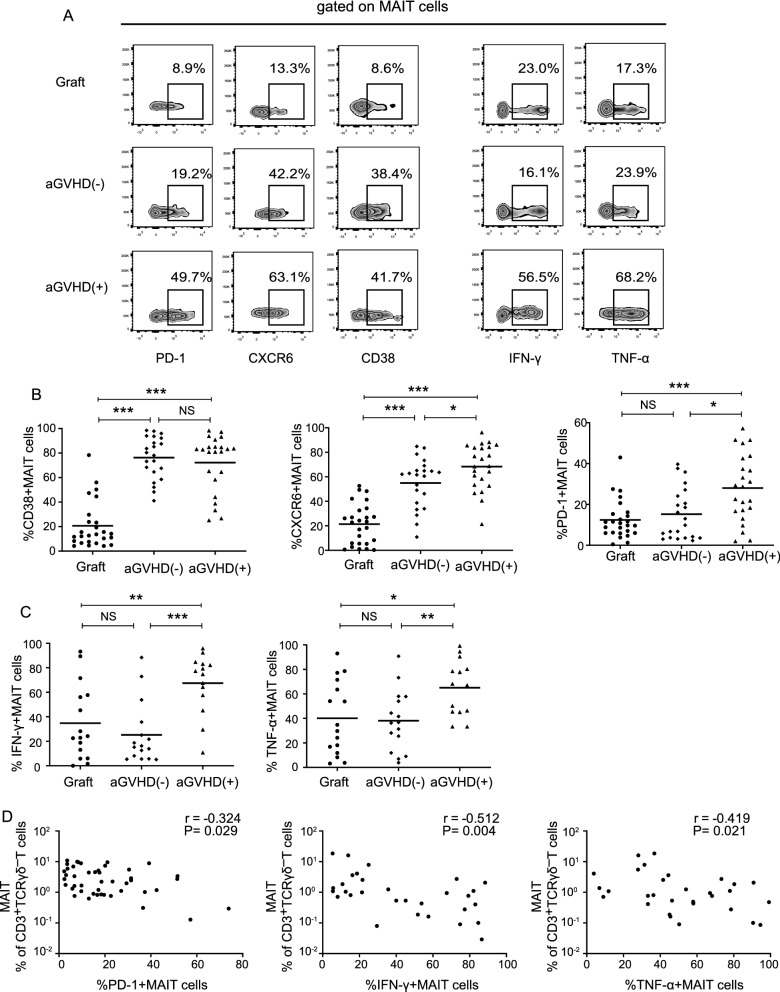
Functional activation of MAIT cells in patients with aGVHD. Representative gating for PD-1, CXCR6, CD38 expression, and IFN-γ, TNF-α production on MAIT cells from PB-grafts, patients without aGVHD and patients with aGVHD development. **B** Expression of PD-1, CXCR6, CD38 on MAIT cells from PB-grafts (n = 26), patients without aGVHD (n = 22) and patients with aGVHD (n = 23). **C** Production of IFN-γ, TNF-α in MAIT cells from PB-grafts (n = 16), patients without aGVHD (n = 16) and patients with aGVHD (n = 14). **D** Spearman rank correlation between circulating MAIT cell frequency and PD-1, IFN-γ and TNF-α expression on MAIT cells from recipients receiving allogeneic HCT. Mean ± standard error is shown. Mann‒Whitney test was used to assess significant differences between groups. *P-value < 0.05; **P-value < 0.01; ***P-value < 0.001; NS: no significance

After analyzing the frequencies and numbers of MAIT cells post allogeneic HCT, we further studied the functional role of MAIT cells in aGVHD development. The MAIT cell activation, migration, and immune response capacity was evaluated through the examination of CD38, CXC chemokine receptor-6 (CXCR6) and programmed death 1 (PD-1) expression levels on MAIT cells [9, 24, 25]. Peripheral blood samples were obtained from 26 PB-grafts, 23 patients with aGVHD, and 22 patients without aGVHD (Fig. 5A). Based on the reported study, we used CD38 as a marker of MAIT cell activation [9]. Our data revealed that the proportion of CD38^+^ MAIT cells were significantly increased in recipient peripheral blood post allogeneic HCT when compared to that in grafts (with aGVHD: 72.3% vs. 20.6%, *P* < 0.001; without aGVHD: 76.2% vs. 20.6%, *P* < 0.001; respectively, Fig. 5B); however, no difference was observed between patients with and without aGVHD (*P* = 0.641). CXCR6 is involved in the trafficking of T cells to peripheral organs such as the liver or intestines [26], and MAIT cells show abundant CXCR6 expression levels [3, 24]. In our study, we found that the circulating MAIT cells exhibited higher levels of CXCR6 in patients with aGVHD than in patients without aGVHD (68.3% vs. 54.9%, *P* = 0.026, Fig. 5B), supporting their migration to the inflammation site and circulating number decrease in aGVHD. In addition, the PD-1 expression was significantly higher in patients with aGVHD (median percentage: 28.0%) than in patients without aGVHD (28.0% vs. 15.2%, *P* = 0.011) and PB-grafts (28.0% vs. 12.4%, *P* < 0.001; Fig. 5B).

We also detected the PMA/ionomycin stimulated cytokine production in MAIT cells from 14 patients with aGVHD, 16 patients without aGVHD, and 16 PB-grafts (Fig. 5A). The IFN-γ^+^ and TNF-α^+^ MAIT cell levels were comparable between the patients without aGVHD and PB-grafts (Fig. 5C); however, we observed a markedly elevated production of IFN-γ and TNF-α in patients who developed aGVHD when compared with patients who did not develop aGVHD (IFN-γ: 67.4% vs. 25.2%, *P* < 0.001; TNF-α: 65.0% vs. 38.1%, *P* = 0.005), as well as the PB-grafts (IFN-γ: 67.4% vs. 34.7%, *P* = 0.006; TNF-α: 65.0% vs. 40.1%, *P* = 0.024; Fig. 5C).

We further used Spearman’s rank test to investigate the correlation between circulating MAIT cell frequencies and their expression levels of IFN-γ, TNF-α, PD-1, CXCR6, and CD38. The frequencies of MAIT cells showed a moderate inverse correlation with PD-1, IFN-γ, and TNF-α expression levels (PD-1: r = ˗0.324, *P* = 0.029; IFN-γ: r = ˗0.512, *P* = 0.004; TNF-α: r = 0.419, *P* = 0.021; Fig. 5D), however, it did not correlate with CXCR6 and CD38 levels. The results demonstrated that MAIT cells from patients with aGVHD had increased inflammatory responses compared to patients without aGVHD and grafts, especially through the IFN-γ and TNF-α activation pathway.

### Transcriptional profile indicated IFN-α response of MAIT cells after HCT

**Fig. 6:**
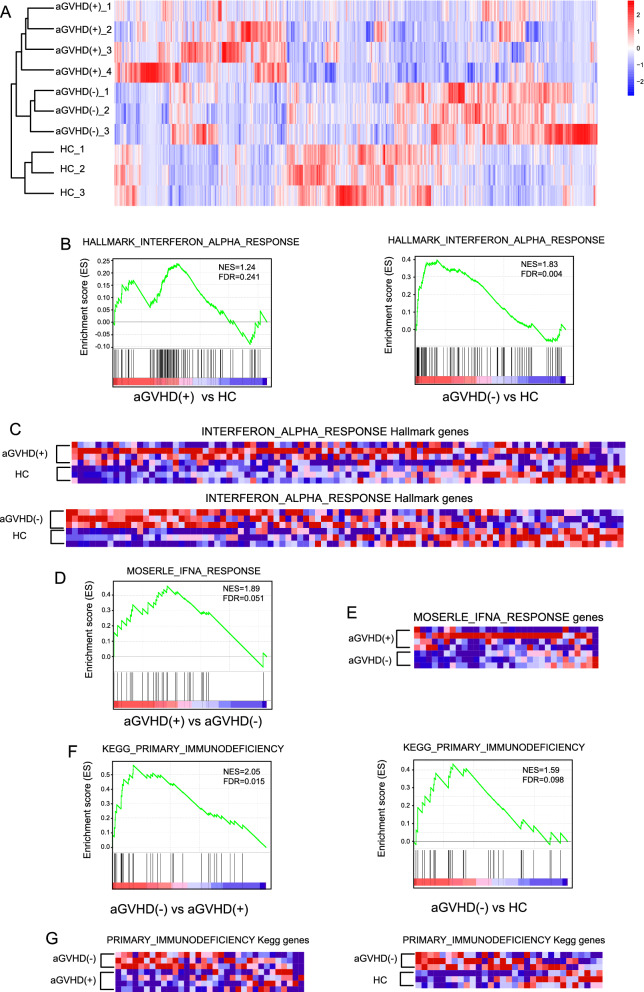
Differential gene expression analysis for MAIT cells from patients and healthy controls. **A** Heatmap of total differential gene expressions. **B** Gene set enrichment analysis (GSEA) for IFN-α response pathway enrichment in MAIT cells from aGVHD patients and non-aGVHD patients when compared to those fron healthy controls (i–ii); Moserle IFN-αresponse pathway enrichment in patients with aGVHD when compared to that in patients without aGVHD (iii). **C** Heatmap presenting the expression of IFN-α-response hallmark genes and Moserle IFN-α-response hallmark genes. **D** GSEA for primary-immunodeficiency gene set in patients without aGVHD when compared with that in patients with aGVHD and healthy controls. **E** Heatmap presenting the expression of primary-immunodeficiency hallmark genes

To identify cell-intrinsic regulatory mechanisms of MAIT cells in aGVHD patients, we performed RNA-seq to identify the transcriptional profile of the circulating MAIT cells from patients with aGVHD (n = 4), patients without aGVHD (n = 3), and healthy controls (n = 3). For the four aGVHD patients, peripheral blood samples were collected when patients were diagnosed with grades C‒D aGVHD, including three cases of grade C aGVHD and one case of grade D aGVHD. Samples from patients without aGVHD were collected at a similar time point according to the collection time from aGVHD patients. There were 754 differentially expressed genes in MAIT cells between patients with and without aGVHD, and 698 genes between aGVHD patients and healthy controls at minimum log (2) fold change of ± 1 (Fig. 6A, Additional file 2). GSEA revealed significant enrichment of interferon-alpha response pathway in patients with aGVHD (NES = 1.24, FDR q-value = 0.241) and patients without aGVHD (NES = 1.83, FDR q-value = 0.004) when compared with healthy controls (Figs. 6B i–ii and 5C i–ii). Some of the shared hub genes with core enrichment in this pathway were *ISG15*, *CD74* and *IRF2* (more details are shown in Additional file 3). Besides, we also observed an enrichment of Moserle IFN-α response gene set between patients with and without aGVHD at a possible trend toward significance (NES = 1.89, FDR q-value = 0.051; Fig. 6B iii and C iii). Some of the hub genes in this gene set were *IFITM1*, *IFIH1*, and *IFIT* family members such as *IFIT5*, *IFIT3*, and *IFIT1*. Additionally, the primary immunodeficiency gene set was enriched in patients without aGVHD when compared with patients who developed aGVHD (NES = 2.05, FDR q-value = 0.015) and healthy controls (NES = 1.59, FDR q-value = 0.098; Fig. 6D). The shared hub genes were *JAK3*, *RAG1*, and *TAP1*.

## Discussion

In this perspective study, our primary objective was to examine the association between MAIT cell reconstitution and aGVHD incidence, including the characteristics of the transfused MAIT cells in grafts. Our data showed that the MAIT cell number appeared as an upward trend within 60 days post allogeneic HCT, however, the MAIT cell proportions reached a plateau and showed a decrease at day 60, partially agreeing with the results of a previous study [17]. Bhattacharyya et al. reported that MAIT cell counts rapidly increased in the first 30 days after HCT and reached a plateau from day 30. Our data showed that from day 30 to 60, the number of MAIT cells was still increasing. Although slightly different, both these studies suggest that MAIT cells could recover rapidly at the early period post HCT. Our research also showed that the type of donor, ATG application and viral infection significantly affected the reconstitution of MAIT cells. Some reports suggest that MAIT cell number in recipients receiving cord blood transplantation is lower than that from bone marrow transplantation or peripheral blood stem cell transplantation, indicating the source of stem cells affects the reconstitution of MAIT cells [18]. In our data, alternative donors, most being haploidentical donors, also affect the reconstitution of MAIT cells. This result may be due to differences in conditioning regimens and GVHD prophylaxis between various stem cell sources, and it was consistent with the characteristics of delayed immune reconstitution in haploidentical transplantation [26]. We also found that viral infections significantly correlate with MAIT reconstitution; however, the causality between them is not clear.

In this study, we longitudinally examined the MAIT cells from peripheral blood stem cell grafts to subsequent peripheral blood samples, which allowed us to systematically investigate the correlation between MAIT cells and aGVHD development. Our data showed that a higher proportion of MAIT cells in the grafts was associated with an increased incidence of aGVHD after allogeneic HCT. However, this result is contrary to a recent study result reported by Gao et al., which showed that lower number of MAIT cells in the graft suggested a higher incidence of intestinal GVHD; however, there was no difference in the incidence of grade I aGVHD or grade II–IV aGVHD [27]. In the study by Gao et al., most patients were simultaneously transfused with peripheral blood stem cells and bone marrow stem cells; however, in our study, patients were only transfused with peripheral blood hematopoietic stem cells. The source of hematopoietic stem cells may partially contribute to the inconsistent results, and the association between MAIT cells in grafts and aGVHD development still needs further clarification. In the process of MAIT cell reconstitution after transplantation, a number of studies, including ours and Gao’s, have shown that the decreased MAIT cells in peripheral blood is related to the increased incidence of aGVHD [17, 18, 27, 28], demonstrating that MAIT cells are involved in the pathological process.

Once activated by antigen recognition or cytokine stimulation, MAIT cells could rapidly release Th1/Th17 proinflammatory cytokines [7, 29]. The critical role of IFN-γ, TNF-α, and IL-17 in the development of aGVHD has been demonstrated [30]. In our report, we found a significantly increased production of these cytokines in circulating MAIT cells in aGVHD patients. Interestingly, no difference was observed in the cytokine secretion levels by MAIT cells between patients without aGVHD and grafts, suggesting the MAIT cell functional activation was critical to the aGVHD development; however, the role of the initiator or amplifier is still unknown. Several studies have shown that MAIT cells exhibiting increased PD-1 expression in infectious diseases and autoimmune diseases are accompanied by a decrease in the MAIT cell number. Thus, we also detected the PD-1 expression level on the surface of MAIT cells, and found similar results in other diseases [13, 31, 32]. PD-1 is a well-known T cell inhibitory molecular, contributing to the exhaustion and anergy of T cells [33]. Blockage of the PD-1/PDL-1 pathway led to aggravation of GVHD symptoms in mice, suggesting a critical role for PD-1 in preventing pathogenic effects of alloreactive T cells [34]. It seems that the increased PD-1 expression on MAIT cells is a negative feedback regulation on the GVHD onset, and subsequently the activation-induced cell death in MAIT cells may contribute to their exhaustion in peripheral blood [35–37]. Taken together, we speculate that when MAIT cells in the donor grafts are transfused into the recipients, they may be activated by cytokines or antigens [7], secreting mass pro-inflammatory cytokines and promote the development of aGVHD. Thus, the increased proportion of MAIT cells in the graft is associated with an increased incidence of aGVHD post allogeneic HCT. With the activation of MAIT cells, negative feedback regulation such as PD-1 is upregulated, the subsequently activation-induced cell death of MAIT cells probably leading to the exhaustion of circulating MAIT cells.

Immunosuppressive treatment is another possible cause affecting MAIT cell frequency and number in peripheral blood. However, so far, some reports reveal that immunosuppressive agents may only have some limited impact on MAIT cells. Sattler et al. reported that immunosuppressive agents in vitro can significantly impair the cytokine production capacity of MAIT cells; however, in patients receiving liver transplantation, reduction of circulating MAIT cells is largely independent of the type and dosage of immunosuppressive agents [38]. In autoimmune hepatitis patients, immunosuppressive treatments also show no impact on circulating MAIT cell proportions [39]. Konuma et al. reported that the administration of calcineurin inhibitors or glucocorticoids had no significant impact on MAIT cell proportion and number in chronic GVHD patients [18]. Our investigation also showed no significant correlation between glucocorticoids cumulative dose and MAIT cell frequency in recipient peripheral blood at day 15 post allogeneic HCT (Additional file 1), indicating that the reduction of MAIT cells is associated with aGVHD development rather than immunosuppressive therapy.

We further explored the expression profile of circulating MAIT cells by RNA-seq and found a significant enrichment of IFN-α-response gene set in recipients receiving allogeneic HCT. Previous reports have demonstrated that MAIT cells could be activated by IFN-α alone [40] or in combination with L-12 or IL-18 in vitro [40, 41]. However, sustained IFN-α stimulation may lead to the dysfunction of MAIT cells [42]. Indeed, in our report, the enrichment of Moserle IFN-α response gene set between recipients with and without aGVHD suggested that a different response to IFN-α may lead to a different outcome. In this study, we also observed an enrichment of primary immunodeficiency gene set in recipients without aGVHD when compared to patients with aGVHD and healthy controls. The hub genes of this gene set were all critical genes involving the T cell signaling (LCK) [43], antigen translocation (TAP1) [44], and immunodeficiency diseases (JAK3 and RAG1) [45]. Based on this immunodeficiency gene set enrichment, we can predict that when MAIT cells show reduced functional activation, similar to the appearance of T cells in immunodeficiency diseases, patients may be less susceptible to aGVHD.

## Conclusions

Our study shows that MAIT cell frequency and the functional status are closely related to the aGVHD development post allogeneic HCT. Meanwhile, MAIT cell activation may also simultaneously induce the exhaustion of MAIT cells, leading to the reduction of MAIT cells in peripheral blood. The IFN-α response perhaps is a critical regulation pathway to the MAIT cell function in aGVHD development. Overall, our data may pave the way for perspective intervention to ameliorate aGVHD.

## Supplementary Information

**Additional file 1:** Other immune cell populations besides MAIT cells in grafts on aGVHD development.

**Additional file 2:** List of differentially expressed gene in RNA-seq.

**Additional file 3:** Hub genes of each enriched gene set.

## Data Availability

The datasets used and/or analyzed during the current study are available from the corresponding author on reasonable request, except the RNA-seq data which are available at Gene Expression Omnibus (GEO) with the accession number of GSE157959.

## Abbreviations

MAIT cells: Mucosal-associated invariant T cells
aGVHD: Acute graft-versus-host-disease
allogeneic HCT: Allogeneic hematopoietic cell transplantation
PMA: Phorbol myristate acetate
TNF-α: Tumor-necrosis-factor-alpha
IFN-γ: Interferon-gamma
IL-17: Interleukin 17
GSEA: Gene set enrichment analysis
ATG: Anti-thymocyte globulin
CXCR6: CXC chemokine receptor-6
PD-1: Programmed death 1
