# Supplementary material for: Mucosal-associated invariant T cells predict increased acute graft-versus-host-disease incidence in patients receiving allogeneic hematopoietic stem cell transplantation
Source: Cancer Cell Int. 2022 Sep 30;22:297. doi: 10.1186/s12935-022-02703-x (PMC9526319; doi:10.1186/s12935-022-02703-x)
Supplement: Supplementary file 3 — Additional file 3: Hub genes of each enriched gene set. [file 12935_2022_2703_MOESM3_ESM.docx]

1. GSEA for HALLMARK_INTERFERON_ALPHA_RESPONSE gene set in aGVHD(+) vs HC.

| Gene | Rank in gene list | Rank metric score | Running ES | Core enrichment |
| --- | --- | --- | --- | --- |
| CCRL2 | 312 | 1.096 | 0.018 | Yes |
| IFI27 | 314 | 1.093 | 0.047 | Yes |
| NMI | 437 | 0.998 | 0.069 | Yes |
| ISG20 | 707 | 0.886 | 0.083 | Yes |
| PARP14 | 950 | 0.834 | 0.096 | Yes |
| SAMD9 | 1464 | 0.747 | 0.097 | Yes |
| EPSTI1 | 1498 | 0.740 | 0.115 | Yes |
| GMPR | 1585 | 0.720 | 0.131 | Yes |
| EIF2AK2 | 1594 | 0.720 | 0.150 | Yes |
| OASL | 2259 | 0.625 | 0.142 | Yes |
| TRIM25 | 2654 | 0.580 | 0.143 | Yes |
| NCOA7 | 2691 | 0.574 | 0.157 | Yes |
| IFITM2 | 2808 | 0.563 | 0.167 | Yes |
| IFIH1 | 3216 | 0.528 | 0.166 | Yes |
| RSAD2 | 3518 | 0.519 | 0.169 | Yes |
| ISG15 | 6458 | 0.497 | 0.071 | Yes |
| PSME2 | 6556 | 0.489 | 0.080 | Yes |
| MOV10 | 6776 | 0.471 | 0.085 | Yes |
| IFITM3 | 7017 | 0.455 | 0.088 | Yes |
| IFI44 | 7116 | 0.447 | 0.096 | Yes |
| CASP1 | 7581 | 0.413 | 0.090 | Yes |
| NUB1 | 7590 | 0.412 | 0.101 | Yes |
| WARS | 7625 | 0.410 | 0.110 | Yes |
| PARP12 | 7780 | 0.398 | 0.115 | Yes |
| PARP9 | 8060 | 0.378 | 0.115 | Yes |
| SAMD9L | 8112 | 0.374 | 0.123 | Yes |
| IRF2 | 8189 | 0.369 | 0.130 | Yes |
| IFITM1 | 8300 | 0.362 | 0.135 | Yes |
| CD74 | 8370 | 0.357 | 0.142 | Yes |
| ELF1 | 8393 | 0.356 | 0.151 | Yes |
| IL15 | 8445 | 0.352 | 0.159 | Yes |
| RTP4 | 8518 | 0.347 | 0.165 | Yes |
| IFI44L | 8641 | 0.338 | 0.170 | Yes |
| IL7 | 8847 | 0.323 | 0.171 | Yes |
| LAP3 | 8907 | 0.319 | 0.177 | Yes |
| LGALS3BP | 8998 | 0.313 | 0.182 | Yes |
| TRIM21 | 9086 | 0.307 | 0.187 | Yes |
| UBE2L6 | 9236 | 0.298 | 0.189 | Yes |
| TXNIP | 9375 | 0.287 | 0.192 | Yes |
| DDX60 | 9383 | 0.287 | 0.199 | Yes |
| TRAFD1 | 9443 | 0.283 | 0.205 | Yes |
| MX1 | 9516 | 0.278 | 0.209 | Yes |
| SELL | 9542 | 0.277 | 0.216 | Yes |
| IRF7 | 9691 | 0.268 | 0.217 | Yes |
| SP110 | 9857 | 0.258 | 0.218 | Yes |
| PLSCR1 | 9970 | 0.251 | 0.221 | Yes |
| C1S | 10088 | 0.243 | 0.223 | Yes |
| IFI35 | 10259 | 0.231 | 0.222 | Yes |
| IFIT2 | 10397 | 0.222 | 0.223 | Yes |
| IL4R | 10491 | 0.216 | 0.225 | Yes |
| PSME1 | 10526 | 0.213 | 0.230 | Yes |
| IFIT3 | 10699 | 0.201 | 0.229 | Yes |
| GBP4 | 10890 | 0.190 | 0.227 | Yes |
| ADAR | 10991 | 0.183 | 0.228 | Yes |
| LY6E | 10992 | 0.183 | 0.233 | Yes |
| BST2 | 11030 | 0.180 | 0.236 | Yes |
| LPAR6 | 11215 | 0.168 | 0.234 | No |
| GBP2 | 11328 | 0.160 | 0.234 | No |
| CASP8 | 11872 | 0.124 | 0.217 | No |
| PSMB9 | 12112 | 0.109 | 0.210 | No |
| PSMA3 | 12274 | 0.098 | 0.207 | No |
| TMEM140 | 12509 | 0.083 | 0.200 | No |
| CMPK2 | 12554 | 0.080 | 0.201 | No |
| USP18 | 12556 | 0.080 | 0.203 | No |
| TRIM26 | 12894 | 0.057 | 0.192 | No |
| SLC25A28 | 13146 | 0.040 | 0.183 | No |
| IRF9 | 13762 | -0.004 | 0.160 | No |
| LAMP3 | 14559 | -0.055 | 0.132 | No |
| DHX58 | 14745 | -0.067 | 0.126 | No |
| RNF31 | 15203 | -0.097 | 0.112 | No |
| STAT2 | 15280 | -0.101 | 0.112 | No |
| CSF1 | 15683 | -0.129 | 0.100 | No |
| RIPK2 | 16229 | -0.174 | 0.084 | No |
| CNP | 16429 | -0.189 | 0.081 | No |
| PSMB8 | 16879 | -0.223 | 0.070 | No |
| TRIM5 | 16942 | -0.229 | 0.074 | No |
| HERC6 | 17310 | -0.257 | 0.067 | No |
| PNPT1 | 17649 | -0.285 | 0.062 | No |
| HLA-C | 18523 | -0.357 | 0.039 | No |
| MVB12A | 18613 | -0.365 | 0.045 | No |
| B2M | 18905 | -0.388 | 0.045 | No |
| TRIM14 | 20238 | -0.515 | 0.008 | No |
| TAP1 | 20700 | -0.562 | 0.006 | No |
| CXCL10 | 22392 | -0.577 | -0.043 | No |
| TDRD7 | 23657 | -0.655 | -0.073 | No |
| PROCR | 23903 | -0.691 | -0.064 | No |
| HELZ2 | 24082 | -0.722 | -0.051 | No |
| CD47 | 24383 | -0.776 | -0.041 | No |
| IFI30 | 24493 | -0.796 | -0.024 | No |
| OGFR | 24525 | -0.803 | -0.004 | No |
| IRF1 | 25033 | -0.932 | 0.002 | No |
| CMTR1 | 25385 | -1.032 | 0.016 | No |
| UBA7 | 25386 | -1.032 | 0.044 | No |

1. GSEA for HALLMARK_INTERFERON_ALPHA_RESPONSE gene set in aGVHD(-) vs HC.

| Gene | Rank in gene list | Rank metric score | Running ES | Core enrichment |
| --- | --- | --- | --- | --- |
| LGALS3BP | 127 | 1.873 | 0.027 | Yes |
| SELL | 248 | 1.584 | 0.049 | Yes |
| PSME2 | 260 | 1.567 | 0.075 | Yes |
| IFI35 | 265 | 1.559 | 0.102 | Yes |
| CSF1 | 325 | 1.462 | 0.124 | Yes |
| IFITM3 | 383 | 1.399 | 0.146 | Yes |
| IFITM2 | 388 | 1.397 | 0.170 | Yes |
| CD74 | 482 | 1.312 | 0.188 | Yes |
| PSMB9 | 504 | 1.292 | 0.209 | Yes |
| CASP1 | 522 | 1.280 | 0.231 | Yes |
| BST2 | 668 | 1.183 | 0.245 | Yes |
| NMI | 958 | 1.120 | 0.253 | Yes |
| IFI27 | 1007 | 1.098 | 0.270 | Yes |
| UBE2L6 | 1190 | 1.048 | 0.280 | Yes |
| IRF2 | 1233 | 1.037 | 0.296 | Yes |
| PARP14 | 1324 | 1.012 | 0.310 | Yes |
| TRIM14 | 1394 | 0.990 | 0.324 | Yes |
| GBP4 | 1518 | 0.957 | 0.335 | Yes |
| SAMD9L | 1590 | 0.939 | 0.349 | Yes |
| ISG15 | 1651 | 0.926 | 0.362 | Yes |
| MVB12A | 1761 | 0.902 | 0.373 | Yes |
| PSME1 | 1915 | 0.871 | 0.382 | Yes |
| GBP2 | 2356 | 0.794 | 0.378 | Yes |
| RTP4 | 2645 | 0.748 | 0.380 | Yes |
| TAP1 | 2870 | 0.717 | 0.383 | Yes |
| TMEM140 | 3190 | 0.673 | 0.382 | Yes |
| OASL | 3421 | 0.644 | 0.384 | Yes |
| RSAD2 | 3571 | 0.626 | 0.389 | Yes |
| TRAFD1 | 3639 | 0.619 | 0.396 | Yes |
| IFI30 | 4773 | 0.581 | 0.362 | No |
| BATF2 | 5381 | 0.578 | 0.348 | No |
| LPAR6 | 5874 | 0.570 | 0.338 | No |
| TRIM25 | 5972 | 0.559 | 0.344 | No |
| TRIM21 | 6167 | 0.538 | 0.345 | No |
| ISG20 | 6387 | 0.516 | 0.345 | No |
| PSMB8 | 6606 | 0.494 | 0.345 | No |
| LAP3 | 7094 | 0.449 | 0.334 | No |
| PARP9 | 7238 | 0.436 | 0.335 | No |
| IFIH1 | 7340 | 0.427 | 0.339 | No |
| SP110 | 7377 | 0.424 | 0.344 | No |
| IL7 | 7965 | 0.376 | 0.328 | No |
| SAMD9 | 8349 | 0.346 | 0.318 | No |
| IL15 | 8350 | 0.346 | 0.324 | No |
| IRF7 | 8944 | 0.298 | 0.306 | No |
| IFITM1 | 9583 | 0.244 | 0.285 | No |
| RNF31 | 9585 | 0.243 | 0.289 | No |
| HLA-C | 9944 | 0.215 | 0.279 | No |
| TRIM26 | 9967 | 0.213 | 0.281 | No |
| CXCL10 | 10161 | 0.199 | 0.277 | No |
| WARS | 10165 | 0.199 | 0.281 | No |
| TRIM5 | 10486 | 0.176 | 0.271 | No |
| USP18 | 10543 | 0.171 | 0.272 | No |
| GMPR | 10878 | 0.148 | 0.261 | No |
| B2M | 11232 | 0.121 | 0.249 | No |
| IFIT2 | 11246 | 0.120 | 0.251 | No |
| LY6E | 12140 | 0.055 | 0.216 | No |
| CCRL2 | 12677 | 0.019 | 0.196 | No |
| NUB1 | 12708 | 0.017 | 0.195 | No |
| NCOA7 | 13868 | -0.057 | 0.150 | No |
| OGFR | 13946 | -0.063 | 0.148 | No |
| DDX60 | 14009 | -0.068 | 0.147 | No |
| C1S | 14630 | -0.117 | 0.124 | No |
| STAT2 | 14828 | -0.132 | 0.119 | No |
| CD47 | 14929 | -0.140 | 0.117 | No |
| PSMA3 | 14970 | -0.143 | 0.118 | No |
| IFI44 | 15016 | -0.145 | 0.119 | No |
| PLSCR1 | 15093 | -0.150 | 0.118 | No |
| ADAR | 16305 | -0.242 | 0.075 | No |
| IRF1 | 16372 | -0.247 | 0.076 | No |
| CNP | 16927 | -0.293 | 0.060 | No |
| PROCR | 17388 | -0.330 | 0.047 | No |
| HELZ2 | 17667 | -0.352 | 0.042 | No |
| MOV10 | 17907 | -0.371 | 0.039 | No |
| EPSTI1 | 18134 | -0.392 | 0.037 | No |
| DHX58 | 18517 | -0.425 | 0.029 | No |
| EIF2AK2 | 18818 | -0.454 | 0.025 | No |
| IRF9 | 19215 | -0.494 | 0.018 | No |
| PARP12 | 19327 | -0.504 | 0.022 | No |
| LAMP3 | 19603 | -0.533 | 0.020 | No |
| SLC25A28 | 19649 | -0.536 | 0.027 | No |
| CASP8 | 19746 | -0.548 | 0.033 | No |
| IL4R | 22280 | -0.584 | -0.057 | No |
| IFIT3 | 22531 | -0.619 | -0.056 | No |
| RIPK2 | 22850 | -0.668 | -0.057 | No |
| CMTR1 | 23236 | -0.733 | -0.060 | No |
| UBA7 | 23277 | -0.739 | -0.049 | No |
| CMPK2 | 23743 | -0.829 | -0.053 | No |
| ELF1 | 24027 | -0.898 | -0.049 | No |
| PNPT1 | 24140 | -0.925 | -0.038 | No |
| TDRD7 | 24307 | -0.977 | -0.028 | No |
| IFI44L | 24470 | -1.030 | -0.016 | No |
| MX1 | 24478 | -1.034 | 0.001 | No |
| HERC6 | 24690 | -1.109 | 0.011 | No |
| TXNIP | 24728 | -1.124 | 0.029 | No |

1. GSEA for MOSERIL_IFNA_RESPONSE gene set in aGVHD(+) vs aGVHD(-).

| Gene | Rank in gene list | Rank metric score | Running ES | Core enrichment |
| --- | --- | --- | --- | --- |
| HERC5 | 11 | 1.965 | 0.157 | Yes |
| EPSTI1 | 652 | 0.875 | 0.203 | Yes |
| ZC3HAV1 | 1346 | 0.730 | 0.235 | Yes |
| SAMD9 | 2223 | 0.610 | 0.251 | Yes |
| IFI44L | 2841 | 0.544 | 0.271 | Yes |
| TRIM22 | 3057 | 0.526 | 0.305 | Yes |
| IFIT5 | 3262 | 0.512 | 0.339 | Yes |
| IFIT3 | 4968 | 0.490 | 0.313 | Yes |
| IFI44 | 5885 | 0.471 | 0.316 | Yes |
| MX1 | 5914 | 0.471 | 0.353 | Yes |
| IFIT1 | 6846 | 0.437 | 0.353 | Yes |
| OASL | 7237 | 0.418 | 0.372 | Yes |
| DDX60 | 7412 | 0.405 | 0.397 | Yes |
| CMPK2 | 7671 | 0.386 | 0.419 | Yes |
| IFI16 | 8310 | 0.341 | 0.422 | Yes |
| RSAD2 | 8591 | 0.324 | 0.437 | Yes |
| IFIH1 | 9118 | 0.288 | 0.440 | Yes |
| IFITM1 | 9200 | 0.282 | 0.460 | Yes |
| OAS1 | 10662 | 0.180 | 0.419 | No |
| IFIT2 | 10895 | 0.164 | 0.423 | No |
| OAS2 | 12181 | 0.082 | 0.381 | No |
| USP18 | 12771 | 0.043 | 0.362 | No |
| RTP4 | 13092 | 0.021 | 0.352 | No |
| STAT1 | 13205 | 0.014 | 0.349 | No |
| SAMD9L | 13674 | -0.018 | 0.332 | No |
| CD274 | 15127 | -0.109 | 0.286 | No |
| GBP1 | 15292 | -0.119 | 0.290 | No |
| DDX60L | 15495 | -0.134 | 0.293 | No |
| DDX58 | 15986 | -0.169 | 0.288 | No |
| TNFSF10 | 16345 | -0.196 | 0.290 | No |
| CXCL10 | 25792 | -1.143 | 0.023 | No |

1. GSEA for PRIMARY_IMMUNODEFICIENCY gene set in aGVHD(-) vs HC.

| Gene | Rank in gene list | Rank metric score | Running ES | Core enrichment |
| --- | --- | --- | --- | --- |
| DCLRE1C | 438 | 1.349 | 0.063 | Yes |
| IKBKG | 734 | 1.161 | 0.120 | Yes |
| ADA | 735 | 1.161 | 0.189 | Yes |
| RFXANK | 1485 | 0.962 | 0.217 | Yes |
| BLNK | 1896 | 0.876 | 0.253 | Yes |
| LCK | 2762 | 0.733 | 0.263 | Yes |
| TAP1 | 2870 | 0.717 | 0.301 | Yes |
| IL7R | 3371 | 0.650 | 0.320 | Yes |
| TAP2 | 3457 | 0.640 | 0.355 | Yes |
| RAG1 | 3676 | 0.614 | 0.383 | Yes |
| AIRE | 5804 | 0.577 | 0.333 | Yes |
| JAK3 | 5833 | 0.575 | 0.366 | Yes |
| CD19 | 6062 | 0.549 | 0.390 | Yes |
| UNG | 6204 | 0.534 | 0.416 | Yes |
| IL2RG | 6516 | 0.503 | 0.434 | Yes |
| CD3D | 8259 | 0.353 | 0.386 | No |
| CD79A | 8321 | 0.348 | 0.404 | No |
| RFXAP | 8703 | 0.318 | 0.408 | No |
| BTK | 12458 | 0.033 | 0.263 | No |
| CD3E | 12766 | 0.013 | 0.252 | No |
| ICOS | 13218 | -0.016 | 0.235 | No |
| RFX5 | 13341 | -0.022 | 0.231 | No |
| PTPRC | 14033 | -0.070 | 0.208 | No |
| ZAP70 | 14821 | -0.131 | 0.185 | No |
| CD40LG | 17791 | -0.361 | 0.090 | No |
| CD4 | 17861 | -0.367 | 0.109 | No |
| TNFRSF13C | 18125 | -0.391 | 0.122 | No |
| CD40 | 19883 | -0.566 | 0.086 | No |
| CIITA | 22558 | -0.623 | 0.018 | No |
| CD8A | 22724 | -0.650 | 0.050 | No |
| CD8B | 24272 | -0.966 | 0.047 | No |

1. GSEA for PRIMARY_IMMUNODEFICIENCY gene set in aGVHD(-) vs aGVHD(+).

| Gene | Rank in gene list | Rank metric score | Running ES | Core enrichment |
| --- | --- | --- | --- | --- |
| RAG1 | 139 | 1.719 | 0.084 | Yes |
| LCK | 500 | 1.225 | 0.133 | Yes |
| TAP1 | 599 | 1.149 | 0.189 | Yes |
| CD19 | 660 | 1.123 | 0.245 | Yes |
| RFXANK | 898 | 1.050 | 0.290 | Yes |
| UNG | 1639 | 0.872 | 0.307 | Yes |
| BLNK | 1669 | 0.866 | 0.351 | Yes |
| IKBKG | 1775 | 0.845 | 0.391 | Yes |
| CIITA | 1844 | 0.835 | 0.431 | Yes |
| CD3D | 2076 | 0.804 | 0.464 | Yes |
| RFXAP | 2897 | 0.679 | 0.468 | Yes |
| ZAP70 | 3055 | 0.659 | 0.497 | Yes |
| BTK | 3649 | 0.588 | 0.505 | Yes |
| CD3E | 3696 | 0.583 | 0.533 | Yes |
| JAK3 | 3726 | 0.579 | 0.562 | Yes |
| IL2RG | 6065 | 0.492 | 0.499 | No |
| PTPRC | 6703 | 0.463 | 0.498 | No |
| DCLRE1C | 7031 | 0.431 | 0.508 | No |
| IL7R | 7887 | 0.356 | 0.494 | No |
| CD79A | 8333 | 0.321 | 0.494 | No |
| AIRE | 10112 | 0.192 | 0.437 | No |
| RFX5 | 10459 | 0.166 | 0.432 | No |
| CD40LG | 10579 | 0.157 | 0.436 | No |
| TAP2 | 12199 | 0.052 | 0.377 | No |
| ICOS | 15315 | -0.150 | 0.267 | No |
| TNFRSF13C | 16557 | -0.238 | 0.232 | No |
| ADA | 16817 | -0.256 | 0.235 | No |
| CD4 | 17561 | -0.306 | 0.223 | No |
| CD8B | 17982 | -0.333 | 0.224 | No |
| CD8A | 18768 | -0.388 | 0.215 | No |
| CD40 | 20285 | -0.465 | 0.181 | No |
| AICDA | 20990 | -0.484 | 0.180 | No |
| TNFRSF13B | 22390 | -0.497 | 0.152 | No |
